# Commentary: sex difference differences? A reply to Constantino

**DOI:** 10.1186/s13229-016-0093-9

**Published:** 2016-06-29

**Authors:** Daniel S. Messinger, Gregory S. Young, Sara Jane Webb, Sally Ozonoff, Susan E. Bryson, Alice Carter, Leslie Carver, Tony Charman, Katarzyna Chawarska, Suzanne Curtin, Karen Dobkins, Irva Hertz-Picciotto, Ted Hutman, Jana M. Iverson, Rebecca Landa, Charles A. Nelson, Wendy L. Stone, Helen Tager-Flusberg, Lonnie Zwaigenbaum

**Affiliations:** University of Miami, Coral Gables, USA; University of California, Davis, Davis, USA; Seattle Children’s Research Institute, Seattle, USA; University of Washington, Seattle, USA; Izaak Walton Killam Health Centre, Halifax, Canada; Dalhousie University, Halifax, Canada; University of Massachusetts, Boston, Boston, USA; University of California, San Diego, La Jolla, USA; King’s College London, London, UK; Yale University School of Medicine, New Haven, USA; University of Calgary, Calgary, Canada; University of California, Los Angeles, Los Angeles, USA; University of Pittsburgh, Pittsburgh, USA; Kennedy Krieger Institute, Baltimore, USA; John Hopkins School of Medicine, Baltimore, USA; Harvard Medical School, Boston, USA; Harvard Graduate School of Education, Cambridge, USA; Boston Children’s Hospital, Boston, USA; Boston University, Boston, USA; University of Alberta, Edmonton, Canada

**Keywords:** Female protective effect, Sex differences, High-risk siblings, Autism spectrum disorder

## Abstract

Messinger et al. found a 3.18 odds ratio of male to female ASD recurrence in 1241 prospectively followed high-risk (HR) siblings. Among high-risk siblings (with and without ASD), as well as among 583 low-risk controls, girls exhibited higher performance on the Mullen Scales of Early Learning, as well as lower restricted and repetitive behavior severity scores on the Autism Diagnostic Observation Schedule (ADOS) than boys. That is, female-favoring sex differences in developmental performance and autism traits were evident among low-risk and non-ASD high-risk children, as well as those with ASD. Constantino (Mol Autism) suggests that sex differences in categorical ASD outcomes in Messinger et al. should be understood as a female protective effect. We are receptive to Constantino’s (Mol Autism) suggestion, and propose that quantitative sex differences in autism-related features are keys to understanding this female protective effect.

## Background

Prospective studies of the high-risk siblings of children with ASD offer an opportunity to examine both sex differences in ASD occurrence and sex differences in ASD traits and related cognitive characteristics. Messinger et al. [1] reported on a large sample (1241 high-risk siblings and 583 low-risk children) recruited at a mean age of 7.25 months, whose cognitive/developmental performance on the Mullen Scales of Early Learning (MSEL) and autism severity scores on the Autism Diagnostic Observation Schedule (ADOS) were assessed at 24 and 36 months. ASD outcome (assessed at 36 months) required both a clinical best estimate diagnosis of ASD as well as an ADOS severity score ≥4.

The female protective effect may be operationalized with respect to the Carter effect, which holds that siblings of female ASD probands will evidence greater ASD affectation (more severe autism traits) than siblings of male probands [2, 3]. This argument has been buttressed by findings of increased genetic liability (e.g., deleterious copy number variants and single-nucleotide variants) in female ASD probands [4]. However, Messinger et al. found that the high-risk siblings of female ASD probands did not differ significantly in ASD outcome, Mullen performance, or severity scores from siblings of male ASD probands. In other words, proband sex was not consequential for the younger high-risk sibling’s outcome.

**Fig. 1:**
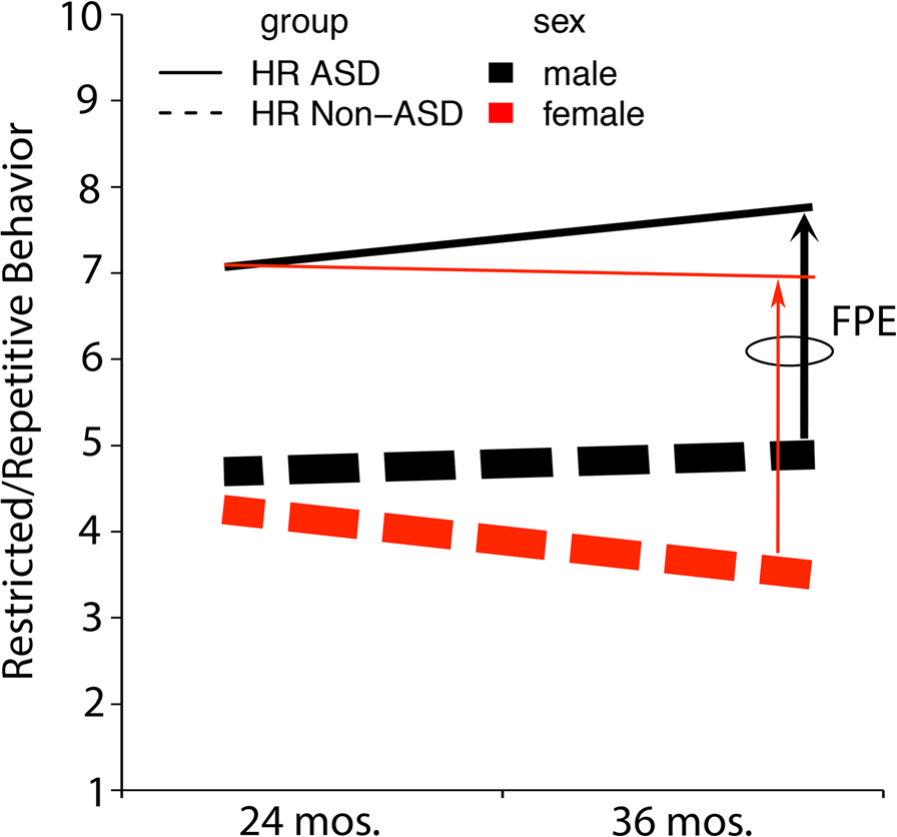
ASD symptoms and ASD recurrence by sex in high-risk siblings with and without ASD. *Horizontal lines* indicate differences between male and female high-risk siblings in ADOS Restricted and Repetitive Behavior (RRB) severity scores [1]. The *vertical arrows* connect high-risk No-ASD and to high-risk ASD groups (see Constantino, 2016, Figure 1). The widths of the *upward arrows* reflect the proportion of high-risk males (.27) and the proportion of HR females (.11) with an ASD outcome. These proportions are also reflected in the width of the *horizontal lines* of male and female ASD groups

Constantino suggests that sex differences in categorical ASD clinical outcomes among the high-risk siblings themselves should be understood as a female protective effect [5]. We are open to this formulation. Quantifying sex differences in categorical ASD outcome is important to understanding the female protective effect. The male to female odds ratio for ASD outcome in Messinger et al. was 3.18, similar to that reported in population-based studies of affected children [6, 7]. The accompanying commentary ([5], see Fig. 1) noted that there were over three times more ASD outcomes among male (193) than female (59) HR siblings. This is correct, but does not reflect the higher absolute number of male HR siblings. The proportion of HR siblings with ASD outcomes was .27 (193/714) for males and .11 (59/527) for females (see Fig. 1).

Messinger et al. found that the under-representation of female categorical ASD outcomes was accompanied by female-favoring sex differences in quantitatively distributed ASD-relevant behaviors [1]. Figure 1 displays restricted and repetitive behavior severity scores from the ADOS, which provide a context for visualizing differences among high-risk siblings in categorical ASD outcomes. Among HR-ASD and HR No-ASD siblings, as well as among LR infants, girls exhibited both higher levels of cognitive/developmental performance and lower levels of restricted and repetitive behaviors than boys. Sex differences, while smaller than the differences between high-risk siblings with and without ASD, were characterized by medium to large effect sizes. An absence of group by sex interaction effects indicated that sex differences in children with ASD were not autism specific; they were also evident in high-risk siblings without ASD and low-risk children.

Overall decrements in quantitative indices of autistic traits among females are common in high-risk siblings with and without ASD [8–11] as well as in the general population [12]. It is likely that population-level reductions in ASD-relevant traits among females lower the propensity for ASD occurrence among females. While certainly not a full explanation for higher rates of ASD among males, sex differences in ASD-relevant traits are pertinent to and, in fact, may be manifestations of a female protective effect.

## Conclusions

The relationship between sex differences in quantitative autistic traits and categorical ASD outcomes involves examination of commonalities and differences in the presentation and causes of ASD in males and females [13–15]. While quantitative and categorical outcomes were not independent in Messinger et al., the large scale prospective results indicate both a robust female advantage in most but not all autism-related traits, and a threefold reduction in female ASD outcomes. Ultimately, a female protective effect manifested in a lower rate of categorical ASD outcomes must be informed by a thorough understanding of female-favoring sex differences in quantitative ASD traits.

## Abbreviations

ADOS, autism diagnostic observation schedule; ASD, autism spectrum disorder; BSRC, Baby Siblings Research Consortium; HR No-ASD, high-risk group without ASD outcomes; HR, high-risk group; HR-ASD, high-risk group with ASD outcomes; LR, low risk group; MSEL, Mullen scales of early learning; RRB, Restricted and Repetitive Behavior Domain Calibrated Severity Score from the Autism Diagnostic Observation Schedule
